# Investigation of Retinal Morphology Alterations Using Spectral Domain Optical Coherence Tomography in a Mouse Model of Retinal Branch and Central Retinal Vein Occlusion

**DOI:** 10.1371/journal.pone.0119046

**Published:** 2015-03-16

**Authors:** Andreas Ebneter, Cavit Agca, Chantal Dysli, Martin S. Zinkernagel

**Affiliations:** Department of Ophthalmology, Bern University Hospital, Inselspital, and University of Bern, Bern, Switzerland; Justus-Liebig-University Giessen, GERMANY

## Abstract

Retinal vein occlusion is a leading cause of visual impairment. Experimental models of this condition based on laser photocoagulation of retinal veins have been described and extensively exploited in mammals and larger rodents such as the rat. However, few reports exist on the use of this paradigm in the mouse. The objective of this study was to investigate a model of branch and central retinal vein occlusion in the mouse and characterize *in vivo* longitudinal retinal morphology alterations using spectral domain optical coherence tomography. Retinal veins were experimentally occluded using laser photocoagulation after intravenous application of Rose Bengal, a photo-activator dye enhancing thrombus formation. Depending on the number of veins occluded, variable amounts of capillary dropout were seen on fluorescein angiography. Vascular endothelial growth factor levels were markedly elevated early and peaked at day one. Retinal thickness measurements with spectral domain optical coherence tomography showed significant swelling (p<0.001) compared to baseline, followed by gradual thinning plateauing two weeks after the experimental intervention (p<0.001). Histological findings at day seven correlated with spectral domain optical coherence tomography imaging. The inner layers were predominantly affected by degeneration with the outer nuclear layer and the photoreceptor outer segments largely preserved. The application of this retinal vein occlusion model in the mouse carries several advantages over its use in other larger species, such as access to a vast range of genetically modified animals. Retinal changes after experimental retinal vein occlusion in this mouse model can be non-invasively quantified by spectral domain optical coherence tomography, and may be used to monitor effects of potential therapeutic interventions.

## Introduction

Retinal vein occlusion (RVO) is a potentially blinding disease affecting an estimated 16,4 million people worldwide, and is the most common retinal vascular disease after diabetic retinopathy[1]. RVO can be divided into branch retinal vein occlusion (BRVO) or central retinal vein occlusion (CRVO). Whereas in CRVO the occlusion occurs at the optic disc affecting all tributaries of the central retinal vein[2], arterial compression of a branch vein is thought to lead to thrombus formation in BRVO[3]. Both CRVO and BRVO result in capillary dropout, and in some cases generalized inner retinal ischemia[4]. Despite the relatively high prevalence there is substantial controversy surrounding the exact patho-mechanisms occurring in RVO and some of the presumed risk factors[5]. Whereas anti-vascular endothelial growth factor treatment has revolutionized the treatment of vascular endothelial growth factor (VEGF) driven macular edema as the main vision threatening sequelae of RVO[6], the causal pathology is not addressed and intravitreal injections are often required frequently and over long periods of time. More instrumental but investigational approaches, such as isovolemic hemodilution[7] or laser-induced chorioretinal venous anastomosis[8], aim at preventing adverse outcomes of RVO by facilitating reperfusion. However, the effectiveness and clinical relevance of the latter treatments remain controversial[9]. The optimal treatment of retinal ischemia and the role of collaterals remain ambiguous[10]. More recent data suggest that inflammation plays an underestimated role in the pathogenesis of RVO[11–13]. The significance of inflammatory processes in the disease pathology can be inferred from the consistent therapeutic effect seen with ocular corticosteroid treatment[14], but little is known about the dynamics and the role of immune cells such as microglia or macrophages in the retina in RVO.

Animal models may help answer some of these questions, and thus facilitate the development of new treatment modalities for BRVO and CRVO. Whereas experimental RVO has been reported in the rhesus monkey[15], pig[16], minipig[17], goat[18], cat[19], dog[20], rabbit[21] and particularly the rat[22–24], there is little data on RVO in mice[25–27]. This is mainly due to the small dimensions of the mouse eye with the ensuing difficulties in manipulation. However, as experimental animals mice have several advantages. Transgenic mice offer unsurpassed possibilities to investigate the role of innate immunity and molecular-biological cascades during RVO. New imaging techniques such as optical coherence tomography (OCT) have only recently become available for retinal imaging in small rodents and allow for longitudinal follow-up in individual animals[28,29]. Here, we characterize a mouse model of RVO using fluorescein angiography (FA) and OCT to quantify retinal changes. Different laser protocols were applied to mimic BRVO and CRVO.

## Materials and Methods

### Animals

This study was approved by the local Animal Ethics Committee (Veterinärdienst des Kantons Bern: BE 38/13) and conformed to the ARVO Statement for the Use of Animals in Ophthalmic and Vision Research. 53 male BALB/c AnNCrl mice (5–6 weeks old, Charles River Laboratories, Sulzfeld, Germany) were housed under temperature and humidity-controlled conditions in individually ventilated cages with a 12-hour light/12-hour dark cycle, and were provided food and water ad libitum. 40 of them were used for OCT follow-up and histology, the remainder of mice was included in the retinal VEGF levels analysis. Inhalation anesthesia with isoflurane (Forene, AbbVie AG, Bar, Switzerland) was used for the laser procedures. For OCT and FA mice were anesthetized by intraperitoneal injection of 1 mg/kg medetomidine (Dormitor 1 mg/mL, Provet AG, Lyssach, Switzerland) and 80 mg/kg ketamine (Ketalar 50mg/ml, Parke-Davis, Zurich, Switzerland). Atipamezol (Antisedan 5 mg/mL, Provet AG, Lyssach, Switzerland) was used to antagonize medetomidine at the end of the intervention. Pupils were dilated with tropicamide 0,5%/phenylephrine 2,5% eye drops (Hospital Pharmacy, Inselspital, Bern, Switzerland). At the end of the experiment, mice were euthanized using 100% carbon dioxide at a fill rate of about 20% to 30% of the chamber volume per minute. Upon completion of the procedure, death was confirmed by ascertaining cardiac and respiratory arrest.

### Laser induced vein occlusion

RVO was induced by 532 nm laser photocoagulation (Visulas 532s, Carl Zeiss Meditec AG, Oberkochen, Germany) using a slit lamp adapter (Iridex Corporation, Mountain View, CA) mounted on a commercially available slit lamp (BM900, Haag-Streit AG, Koeniz, Switzerland) within 3 minutes after intravenous injection of 0.15 ml rose bengal (4,5,6,7-tetrachloro-2',4',5',7'-tetraiodo-fluorescein (Sigma-Aldrich Switzerland, Buchs, Switzerland), 5mg/ml saline) into the tail vein, similar to the protocol employed by others[24,27]. One vein was occluded in the single vein laser group whereas all veins were targeted in the multiple vein laser group. A small 2 mm fundus laser lens (Ocular Instruments, Inc., Bellevue, WA) was used to visualize the fundus and blood vessels during the laser treatment. Hydroxypropylmethylcellulose 2% (Methocel, OmniVision AG, Neuhausen, Switzerland) was used as viscous coupling fluid. The laser settings were 50 μm spot size, 160 mW and 0.8–2.5s. In most eyes several burns were necessary to achieve occlusion of the retinal vessel. Burns were applied sequentially from distal to proximal to minimize the risk of iatrogenic vitreous hemorrhage. On average 2–5 burns were applied per vein. Occlusion was deemed complete when whitening of the blood vessel and stasis in its distal parts were observed on direct fundus observation with the laser lens during the laser procedure. In some animals the central vein occlusion phenotype was noted on subsequent examinations despite targeting only a single vein, most likely because the laser burns were placed too close to the optic nerve or variable susceptibility of different batches of animals. Hence, the final assignment to experimental groups was made based on the phenotype on FA during follow-up.

### Fundus color photography

**Fig 1:**
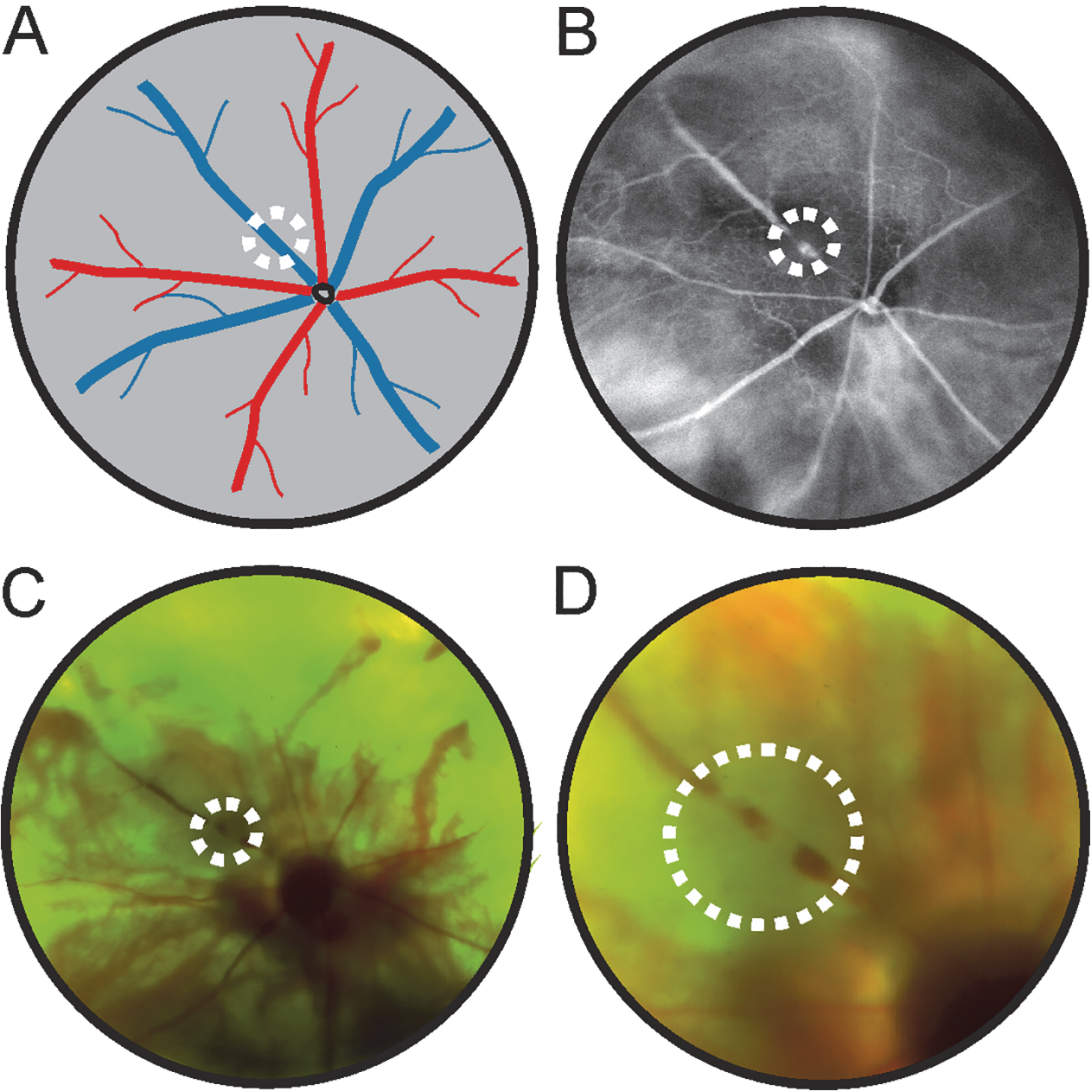
Experimental retinal vein occlusion in mice. (**A**) Location of experimental vein thrombosis. (**B**) Confirmation using ultra-widefield retinal imaging three days after occlusion. The vein appears dilated distally with reduced blood vessel caliber proximal to the intervention site. (**C&D**) Color retinal imaging using ultra-widefield technology illustrating the situation immediately after application of the laser burn. The veins appear white at the occlusion site and stasis of the blood column is observed. (**C**: overview, **D**: laser site at higher magnification).

Vascular occlusion was confirmed and documented immediately after laser application by fundus color photography at different magnifications (Fig. 1C (low magnification), 1D (high magnification)). Images were taken with an ultra-widefield panoramic 200TX device (Optos plc, Dunfermline, Scotland) without a contact lens, as described elsewhere[30].

### Fluorescein angiography

A confocal scanning laser ophthalmoscope (Heidelberg Spectralis HRA+OCT, Heidelberg Engineering GmbH, Heidelberg, Germany) was used for FA using a standard 55° lens without any additional contact lens on the mouse eye. For orientation an infrared image was acquired before subcutaneous injection of 5μg fluorescein (Fluoresceine 10% Faure 0.5g/5ml, Novartis Pharma Schweiz AG, Rotkreuz, Switzerland). Consecutive photographs were taken with late images taken two minutes after the fluorescein injection.

### OCT acquisition

Mouse retina OCTs were acquired using the Heidelberg Spectralis HRA+OCT system according to the protocol described by Fischer et al. with slight modifications. [31] Briefly, a +4 dpt rigid gas permeable contact lens (Quantum I, Bausch + Lomb Inc., Rochester, NY) was placed on the mouse eye, and a 78D standard ophthalmic non-contact slit lamp lens (Volk Optical Inc., Ohio, USA) was taped in front of the standard 30° Heidelberg Spectralis optic. The mouse eye had previously been dilated with tropicamide 0,5%/phenylephrine 2,5% eye drops (Hospital Pharmacy, Inselspital, Bern, Switzerland) and hydroxypropylmethylcellulose 2% (Methocel, OmniVision AG, Neuhausen, Switzerland) was used as coupling gel.

Volume scans centered on the optic nerve were acquired using the ‘fast’ preset in the Automatic Real-Time (ART) mode, averaging 9 frames per image. Each volume covered 20° x 20°, and consisted of 25 horizontal B-scans (512 A-scans each), 240μm apart.

### Retinal thickness measurements using OCT images

The main purpose of OCT imaging was quantification of the total retinal thickness. In the first instance the Heidelberg Eye Explorer (Version 1.7.1.0, Heidelberg Engineering GmbH, Heidelberg, Germany) segmentation algorithm was applied, which recognizes the internal limiting membrane and the basal membrane. Placement of the segmentation lines was checked on all OCT scans by experienced OCT readers (AE, CD). The software reliably identified the internal limiting membrane and manual correction was rarely required here. However, in most eyes the basal membrane segmentation line was placed on the sclera. It was therefore necessary to manually reposition this segmentation line after visual identification of the basal membrane on almost all scans.

For analysis of the retinal thickness a standard ETDRS grid with circle diameters 1mm, 3mm, and 6mm was centered on the optic nerve head. Retinal thickness measurements displayed for the inner ring were recorded in each quadrant (superior, anterior, inferior, posterior). This data was then used for statistical analysis. Of note, lateral measurements are not accurate since the dimensions of the mouse eye differ significantly from a human eye. However, axial OCT measurements seem fairly accurate[32].

### Histology

Retinas from experimental animals were examined histologically seven days after laser induced RVO. The eyes were removed from killed animals and fixed for at least 24 hours in 10% buffered formalin. A corneal suture was used to define the orientation of the eye and identify the lasered area of the retina. Samples were then processed for routine paraffin embedding and 4 μm sections containing the optic nerve head were cut. After deparaffinization, sections were stained with hematoxylin & eosin and permanently mounted. The cell density in the ganglion cell layer was estimated by manually counting the cell nuclei in this layer per unit of length from ora serrata to ora serrata (S1 Fig.). The average inner nuclear layer thickness was approximated in a similar way: Briefly, the area comprising the inner nuclear layer on histology sections was measured using imaging analysis software (ImageJ for Mac OS X version 1.48, National Institute of Health, Bethesda, MD) and then divided by the length of the assessed retinal area. The parts of the retina with signs of collateral damage in the outer layers at the laser site were not included in this quantification.

### VEGF Immunoassay

Retinal VEGF levels after experimental vein occlusion were analyzed by the mouse VEGF Quantikine ELISA immunoassay (R&D Systems, Inc., Minneapolis, MN) according to manufacturer's instructions. Briefly, experimental and control contralateral retinas were isolated at the 1 and 3 day time points. Retinas were triturated in 200 μl phosphate-buffered saline using a polytron for 20 seconds at level 3. The retina suspensions were then centrifuged at 5000 g for 5 minutes. Supernatants were diluted 1:4 using the RD5T diluent from the kit and assayed for VEGF quantification. Absorbance was read for each sample at 450 nm wavelength with reference to 570 nm wavelength using an Infinite M1000 PRO microplate reader (Tecan Group Ltd., Maennedorf, Switzerland). Blank reads were subtracted from the corrected 450 nm reads and a standard curve was generated according to the dilutions of the standard sample (*Sf* 21-expressed recombinant mouse VEGF_164_) provided by the kit.

### Statistics

Results are presented as mean ± SEM. For statistical analysis of retinal thickness on OCT a one-way ANOVA followed by a Tukey’s multiple comparisons test was performed. To assure independence of samples the analysis was conducted for right and left eyes separately because both eyes were included in the study from some animals. A two-way ANOVA with Tukey’s multiple comparisons test was used to analyze VEGF data. For this experiment only one eye per animal had been laser treated and the contralateral eye served as control. The significance level was set at alpha = 0.05. Statistical data analysis was performed using GraphPad Prism version 6 for Mac OS X (GraphPad Software Inc, La Jolla, California, USA).

## Results

57 eyes of 31 mice with bilateral experimental retinal vein occlusion were included for imaging and OCT analysis. 25 eyes had one vein occluded, 32 eyes presented with the central retinal vein occlusion phenotype, and 18 eyes were used as controls. The occurrence of vitreous hemorrhages (n = 4) was the most common reason that eyes had to be excluded from final analysis. Another 26 eyes of 13 animals with unilateral experimental retinal vein occlusion were included for retinal VEGF analysis, the contralateral eye serving as control.

### Fluorescein angiography

**Fig 2:**
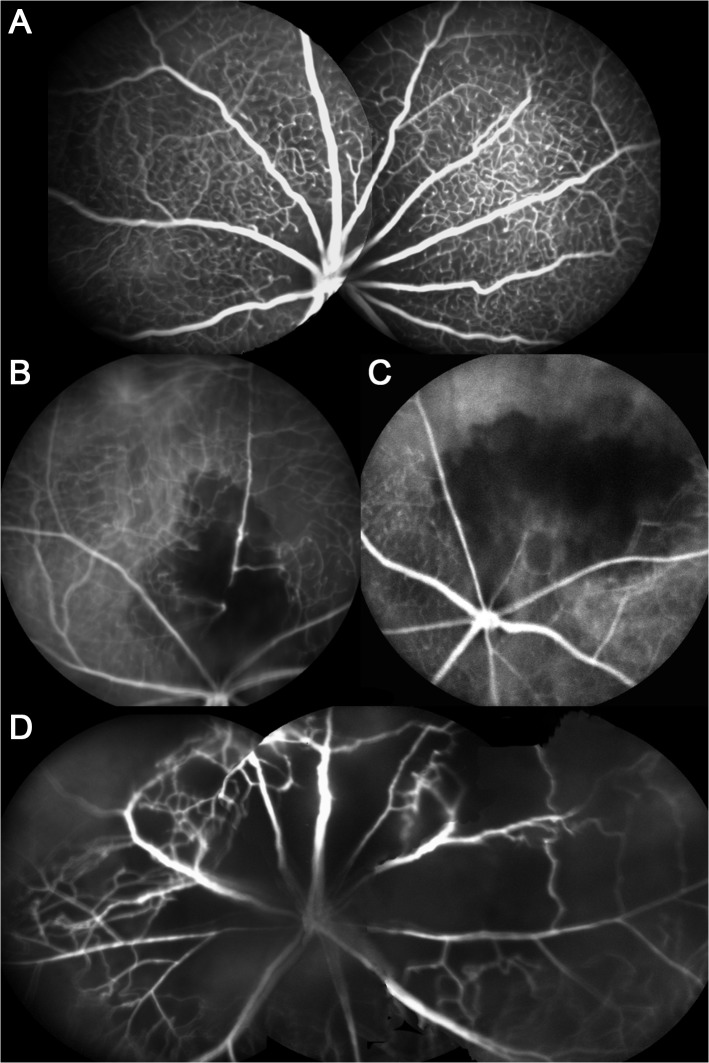
Angiographic findings with different laser patterns seven days after experimental retinal vein occlusion. (**A**) High-resolution fluorescein angiogram (Heidelberg Spectralis HRA) displays the structure of the blood vessels at the level of the capillaries in a control mouse. (**B-D**) Images represent the distinct angiographic patterns after different types of laser application. (**B**) Successful experimental retinal vein occlusion of a single vessel with blood flow still absent proximal to the interventional site. Some drop out of capillaries in the drainage area of the affected vein is observed, which is particularly marked adjacent to the laser site. (**C**) In some experimental eyes the arterial side of the circulation is affected too, and a complete breakdown of capillary perfusion is manifest in a sector of the retina. (**D**) In an experimental animal, which had all veins occluded, capillary drop out is more widespread and affects all quadrants to a similar extent. Some veins show reperfusion.

FA showed clearly distinct areas of capillary non-perfusion distal to the occlusion site. The different laser patterns resulted in two distinctly different phenotypes: Firstly, sectoral capillary drop out to a variable degree was induced by laser application to a single vein about one to two disc diameters from the optic nerve (Fig. 1). The amount of capillary dropout ranged from discrete changes on FA (Fig. 1B) to more discernible sectoral capillary loss (Fig. 2B). Second, occlusion of all retinal veins around the optic nerve head caused more widespread breakdown of capillaries (Fig. 2D) resembling CRVO in humans. No retinal neovascularizations were detected on FA in any eyes during the observational period of up to four weeks.

### Histological changes

**Fig 3:**
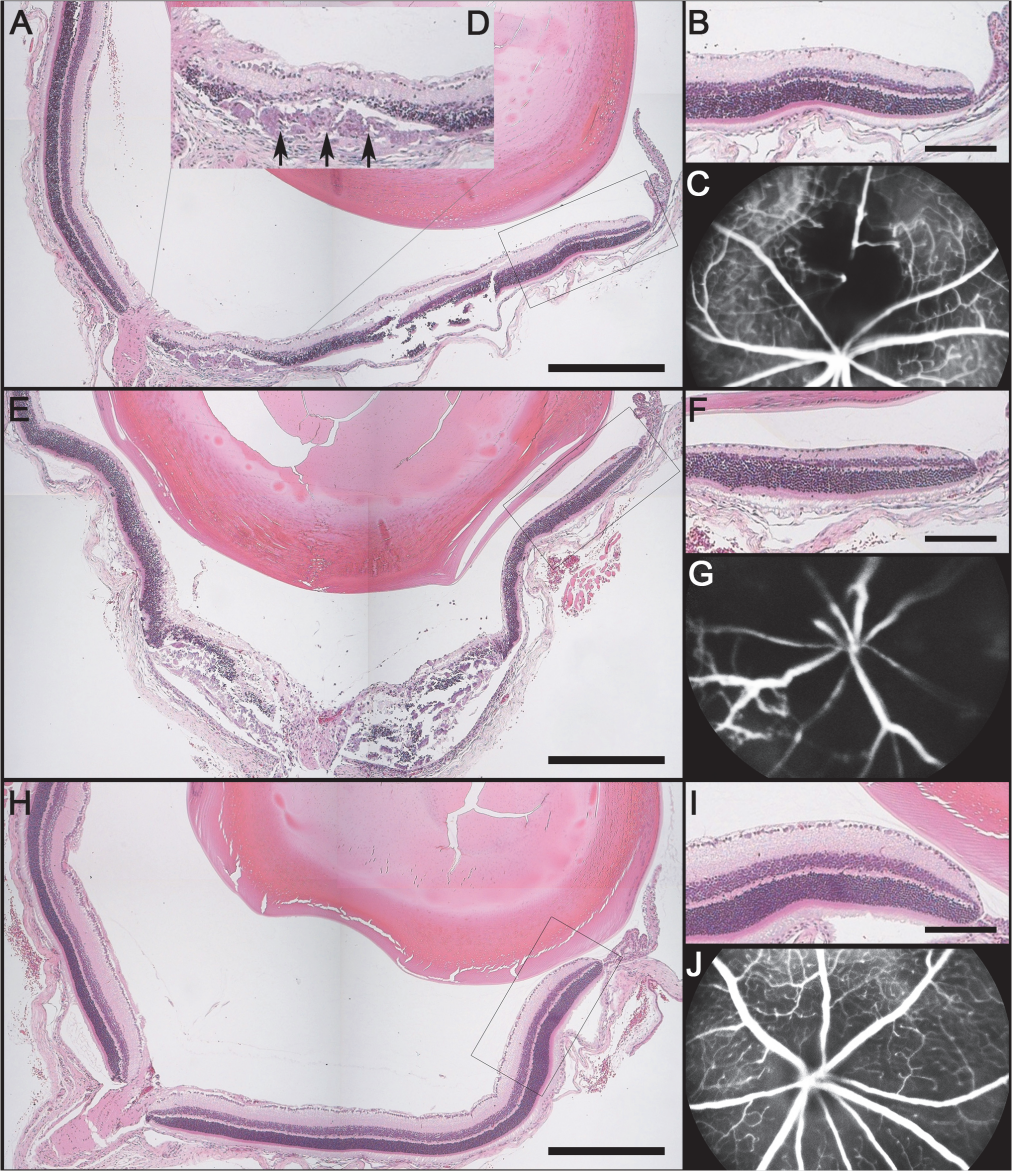
Correlation of angiographic findings with histology seven days after laser photocoagulation. Representative images after (**A**-**D**) single vein occlusion, (**E-G**) multiple vein occlusion, and (**H**-**J**) a control eye. (**A**, **E**, **H**) Whole eye hematoxylin & eosin sections [scale bars 500 μm], (**B**, **F**, **I**) magnified cutouts of the peripheral retina [scale bars 150 μm], and (**C**, **G**, **J**) fluorescein angiograms. At the laser site destruction of the outer retinal layers is evident (inset **D**) with a subretinal scar-like lesion (black arrows), representing collateral damage. (**E**) These changes are more pronounced in eyes where all veins have been occluded. (**B**, **F**) Interestingly, the inner retinal layers distal to the interventional site are thinner than in the control (**I**), whereas the outer nuclear layer and the photoreceptor outer segments are of normal dimensions. The outer plexiform layer is partially missing. The cell density in the ganglion cell layer is reduced in eyes after laser to (**F**) multiple veins and after (**B**) single vein laser in the affected area of the retina.

The histological analysis of eyes with experimentally occluded veins revealed changes, which corroborate the presence of inner retina hypoxia in the sectors affected by venous stasis. The main feature was thinning of the inner two thirds of the retina (Fig. 3A and 3E). All inner retinal layers were affected. The cell density in the ganglion cell layer was reduced (p<0.0001). Thinning of the inner plexiform layer was evident. Likewise, the number of nuclei in the inner nuclear layer was diminished (p<0.0001; S1 Fig.). The outer plexiform layer was completely absent in the more posterior parts of the retina. Interestingly, this layer seemed to be more preserved adjacent to the ora serrata (Fig. 3B and 3F). All these changes appeared slightly more obvious in eyes that had had laser to multiple veins (Fig. 3E).

Collateral damage affecting the photoreceptors was evident in areas adjacent to the laser spots (Fig. 3A and 3E). Additionally, in these zones subretinal lesions (Fig. 3D) were found that had a similar appearance to those described by Chidlow et al. after laser application to the retina[33]. Structural changes were particularly marked in eyes after laser to multiple veins (Fig. 3E).

**Fig 4:**
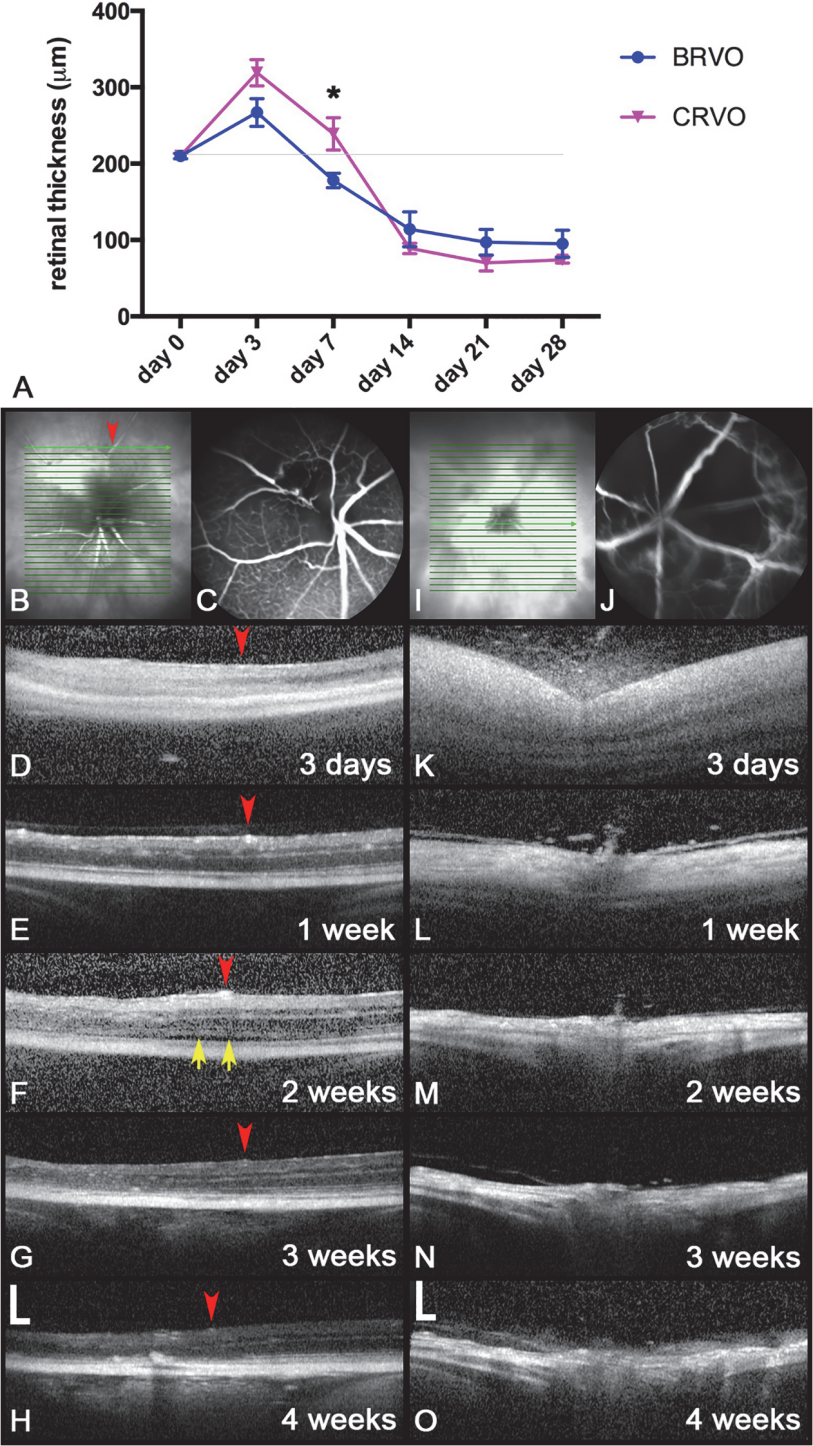
Optical coherence tomography and thickness measurements. (**A**) Mean total retinal thickness (means±SEM) over 4 weeks. The retinal thickening is marginally greater after occlusion of all retinal veins (CRVO) than after blockage of a single vein (BRVO) up to about day 7 (* p = 0.0426). At later time points the retinal thickness is decreased in the affected areas after both CRVO and BRVO, but there is no longer a statistical difference between the two laser patterns. The mean retinal thickness in affected quadrants is significantly different (p < 0.001) from control eyes (grey line) at all time points except day 7. (**B**-**H**) BRVO phenotype after occlusion of a single vein. (**C**) Fluorescein angiogram. (**D**-**H**) Serial scans taken at the location highlighted on photograph (**B**). The red arrowhead marks a retinal artery at the right border of the catchment area of the occluded vein. (**I**-**O**) CRVO phenotype after occlusion of multiple veins. (**J**) Fluorescein angiogram. (**K**-**O**) Horizontal scans taken through the optic nerve head. (**D, K, L**) Hyperreflectivity with washed-out retinal structures and thickening of the retina are observed within the first few days. (**F-H**, **M-O**) Thereafter progressive thinning of the inner retinal layers is evident. (**F**) A sliver of subretinal fluid (yellow arrows) is apparent [scale bars 200 μm].

Cellular elements were also found in the vitreous (Fig. 3A and 3E). They were scattered in the vitreous cavity, and cell density seemed to be more marked neighboring the ora serrata (Fig. 3A, left part of the microphotograph) and the optic nerve head (Fig. 3E). Cellular infiltration of the area above the optic nerve head was particularly evident in eyes that had severe ischemic changes on FA and OCT (Fig. 4K–4M).

### OCT changes

Retinal thickness changes over time were analyzed using the OCT imaging data. For the eyes with single vein laser, the retinal thickness in the treated quadrant had increased significantly (Fig. 4A: BRVO) on day 3. However, at subsequent time points from day 7 onwards the thickness of the retina steadily decreased, and was significantly thinner than in control eyes (218±3.2μm). Interestingly, although there was initial thickening, intraretinal cysts were not observed. In eyes that had occlusion of multiple veins, similarly, a significant increase in retinal thickness was observed on day 3 after laser occlusion (Fig. 4A: CRVO). However, the diffuse retinal edema quickly resolved thereafter, and by day 7 the retinal thickness was back to almost normal values. Subsequently, retinal thinning was noted to a similar extent as in eyes with a single occluded vein. In areas with impaired perfusion the structural features of the retina vanished and the retina appeared hyperreflective on OCT (Fig. 4D, left from the red arrow; Fig. 4K, 4L). Besides the sporadic occurrence of some cellular infiltration of the vitreous above the optic nerve head (Fig. 4K–4M) small amounts of subretinal exudative fluid were occasionally detected (Fig. 4F, yellow arrows).

### Reperfusion patterns in longitudinal follow-up on FA

**Fig 5:**
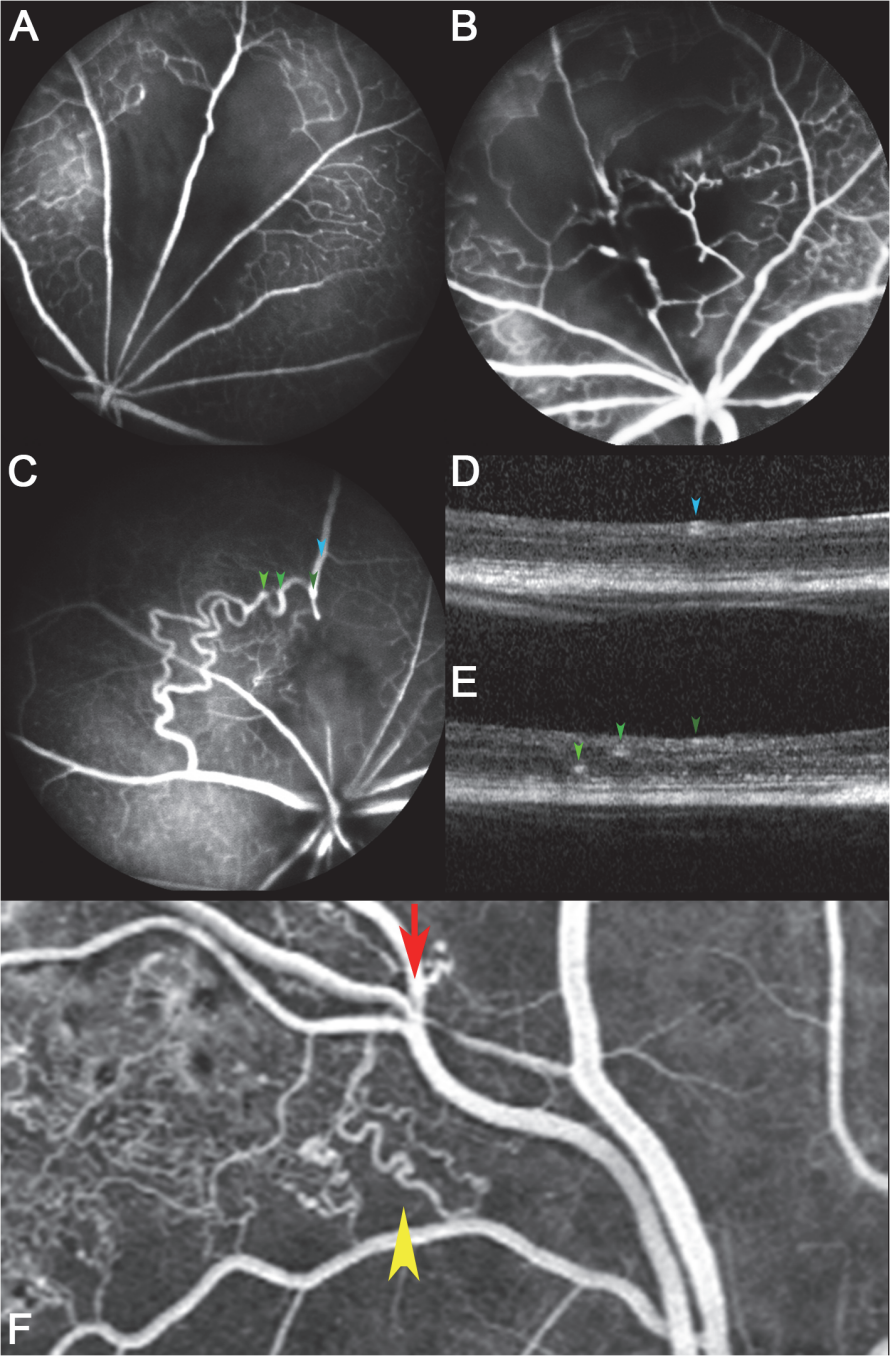
Reperfusion patterns after single retinal vein occlusion. (**A**–**C**) Angiographic findings observed 7–14 days after experimental retinal vein occlusion are displayed. (**A**) Reperfusion had occurred in some eyes at week one. (**B**, **C**) Collateral blood vessels were evident in others. (**C**) These collaterals observed in an eye two weeks after experimental RVO were particularly prominent. (**D**, **E**) The optical coherence tomography scans taken horizontally from this eye highlight the depth location of the different vascular plexuses in the retina. While the normal major retinal veins (blue and dark green arrowhead) lie adjacent to the inner limiting membrane, the collaterals, representing dilated preexisting blood vessels, are found deeper in the retina (light green and green arrowhead in **E**). (**F**) Collateral blood vessels in a human with chronic BRVO for comparison. The vein is blocked at an arterio-venous crossing (red arrow) and appears dilated distal to the occlusion site. Tortuous collateral veins (yellow arrowhead) span from the dilated occluded vein to a proximal venous branch.

Two main reperfusion processes were identified after experimental RVO. Reperfusion of the occluded blood vessel was the most common mechanism (Fig. 5A). Alternatively, collateral vessels developed over time. These were usually present 7 days after the intervention (Fig. 5B) and sometimes became more prominent after longer periods (e.g. 2 weeks in Fig. 5C). The appearance of these blood vessels was very similar to collaterals in humans (Fig. 5F). Interestingly, the collaterals run in all three vascular plexuses[34] of the mouse retina (Fig. 5D, 5E: superficial (dark green arrow), intermediate (green arrow), deep (light green arrow)).

### VEGF

**Fig 6:**
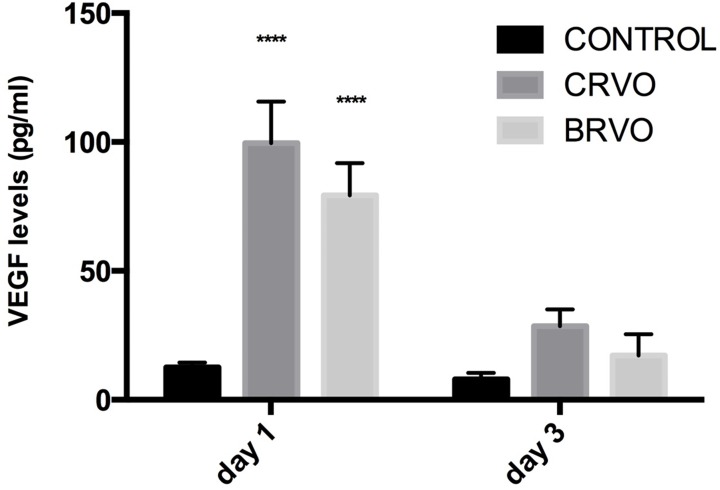
Retinal vascular endothelial growth factor (VEGF) levels. Bar graph of retinal VEGF levels at day 1 and day 3 after experimental vein occlusion (values represent means±SEM, where n = 6 for controls and n = 3 for each experimental group at each time point, **** p < 0.0001). Retinal VEGF levels were raised relative to controls on day 1, but the difference between the CRVO model and the BRVO model was not statistically significant at any time point.

VEGF levels in the retina were 99.6±16.1 pg/ml for the CRVO group and 79.3±12.6 pg/ml for the BRVO group at day 1, compared to 12.5±2.0 pg/ml in the control group (Fig. 6). On day 3 the VEGF levels had decreased to 28.5±6.5 pg/ml in the CRVO group and 17.2±8.3 pg/ml in the BRVO group, respectively. Retinal VEGF levels were significantly different from those in control eyes on day 1 (p<0.0001). The difference between the BRVO and the CRVO group was not significant at these time points.

## Discussion

In humans, the acute phase of partial or complete venous occlusion features ischemia, retinal hemorrhages and edema in the neuroretina. The acute phase is followed by a protracted phase dominated by remodeling of vessels and sometimes development of collateral venous drainage bypassing the site of occlusion. The chronic phase is largely influenced by the degree of ischemia and ensuing complications such as neovascularization. We here present the clinical findings in a highly reproducible mouse model of laser induced RVO. Depending on the number of veins occluded by photothrombosis, the model either mimics the features of CRVO or BRVO. Similar to RVO seen in humans, several stages can be differentiated in the mouse model: Immediately following laser photocoagulation the venous blood flow is interrupted and the veins become slightly dilated and tortuous. In the acute phase there is marked edema of the neuroretina as seen on OCT, followed by atrophy of the inner layers of the retina in the chronic phase. In the current model we have not observed any new vessels, independently of the type of laser application. This is in keeping with the findings reported by other groups[25,27]. Only in a single paper on C57BL/6 were new vessels noted[27] in non-modified mice. In another paper new vessels after experimental RVO in the mouse were only observed after durable hematopoietic stem cell grafting[25]. Depending on the aims of the study, different strains of animals may be favorable. The occlusion rate between pigmented and albino mice seems to be similar[35]. However, in pigmented mice more energy will be absorbed by the RPE and choroid, and the rate of choroidal new vessel formation can be expected to be higher in pigmented animals.

We have performed FA with two different devices, namely an ultra-widefield system (Optos 200Tx) and confocal scanning laser ophthalmoscopy (Heidelberg Spectralis HRA). The latter carries the advantage that it is possible to separately image the three vascular plexuses of the mouse by focusing on the different layers[36]. A robust RVO mouse model may be helpful as there is only limited knowledge of the cascade of events triggered at the cellular level after RVO. As such this model is well suited to study the role of acute inflammatory mediators and local immune cells such as microglia or invading macrophages. Not only has inflammation been connected to thrombus formation[37], but it is even more instrumental in the self perpetuating cycle leading to establishment of chronic macular edema secondary to vein occlusion, which can persist despite collateral formation and subsiding tissue hypoxia[38,39]. Inflammation together with increased intraluminal pressure favors leakage of serum proteins and augmented oncotic pressure in the interstitium. This in turn further impairs capillary perfusion and exacerbates hypoxia, boosting formation of VEGF and inflammatory cytokines[40]. The success of intravitreal steroids for the treatment of macular edema secondary to RVO has shown that inflammation is likely to play a major role in the chronic phases of this disease[14]. However, due to the side effects of this substance class, such as cataract formation or steroid induced glaucoma, a more targeted drug against specific elements in the inflammatory cascade following RVO would be desirable.

There are some limitations to the model presented in this paper. First, cystoid edema was not observed in any of the experimental animals. In humans, leakage from vascular damage of retinal vessel is thought to result mainly in cyst formation in the inner nuclear layers, whereas outer blood-retinal barrier dysfunction is associated with cysts in the Henle fiber layer[41], and sometimes subretinal fluid[42]. Muller cells have a central role in edema formation and can become swollen themselves[43]. The ‘Z-shaped’ morphology of Muller cells is characteristic for the structure of the human fovea[41]. Rodents do not have a macula and lack the characteristic features of the macula in man. The human macula is characterized by high cell density and metabolic activity, and is therefore particularly predisposed for dysfunction if the metabolism is compromised[44]. Furthermore, cyst formation might be a sign of chronicity. A healthy retina can probably compensate for acute damage to the retinal vasculature. These are possible explanations for the fact that we did not observe cystoid changes in edematous retinas after experimental RVO. Another limitation of our model is that the laser spot itself may cause an inflammatory response, which may in turn influence the course of RVO in the mouse.

Despite these limitations this model has several advantages. In the first instance, the same imaging techniques as in humans, FA and OCT, can be used. This is to our knowledge the first longitudinal quantification of retinal morphology in a mouse model of BRVO and CRVO using spectral domain OCT. OCT allows a reliable quantification of retinal swelling and degeneration in the same mouse at different time points, offering an excellent *in vivo* readout for therapeutic agents in preclinical studies. Models of RVO represent ideal tools to study ischemia-reperfusion injury of the central nervous system. The eye as an investigational organ has several advantages over stroke models involving brain damage proper with ensuing morbidity affecting the experimental animal. Due to the transparent nature of the ocular media the retina can be observed *in vivo* in a longitudinal fashion. The neuronal cells in the retina are highly organized and systematic histological evaluation is straightforward.

The current model is not labor intensive, minimally invasive, and does not cause any systemic side effects or morbidity to the mice. Drug delivery is possible via intravitreal administration and not restricted to systemic application. The degree of ischemia is highly reproducible because the catchment area of the individual veins is relatively constant.

## Supporting Information

S1 Fig Cell density in the ganglion cell layer and thickness of the inner nuclear layer in mouse eyes with experimental central retinal vein occlusion. The reduction of the cell density in the ganglion cell layer was 45% (p < 0.0001; n = 6) compared to control eyes (n = 3). The decrease in thickness of the inner nuclear layer was of similar magnitude (47%, p < 0.0001).
(TIF)
