# Supplementary figures and images for: Investigation of Retinal Morphology Alterations Using Spectral Domain Optical Coherence Tomography in a Mouse Model of Retinal Branch and Central Retinal Vein Occlusion
Source: PLoS One. 2015 Mar 16;10(3):e0119046. doi: 10.1371/journal.pone.0119046 (PMC4361633; doi:10.1371/journal.pone.0119046)

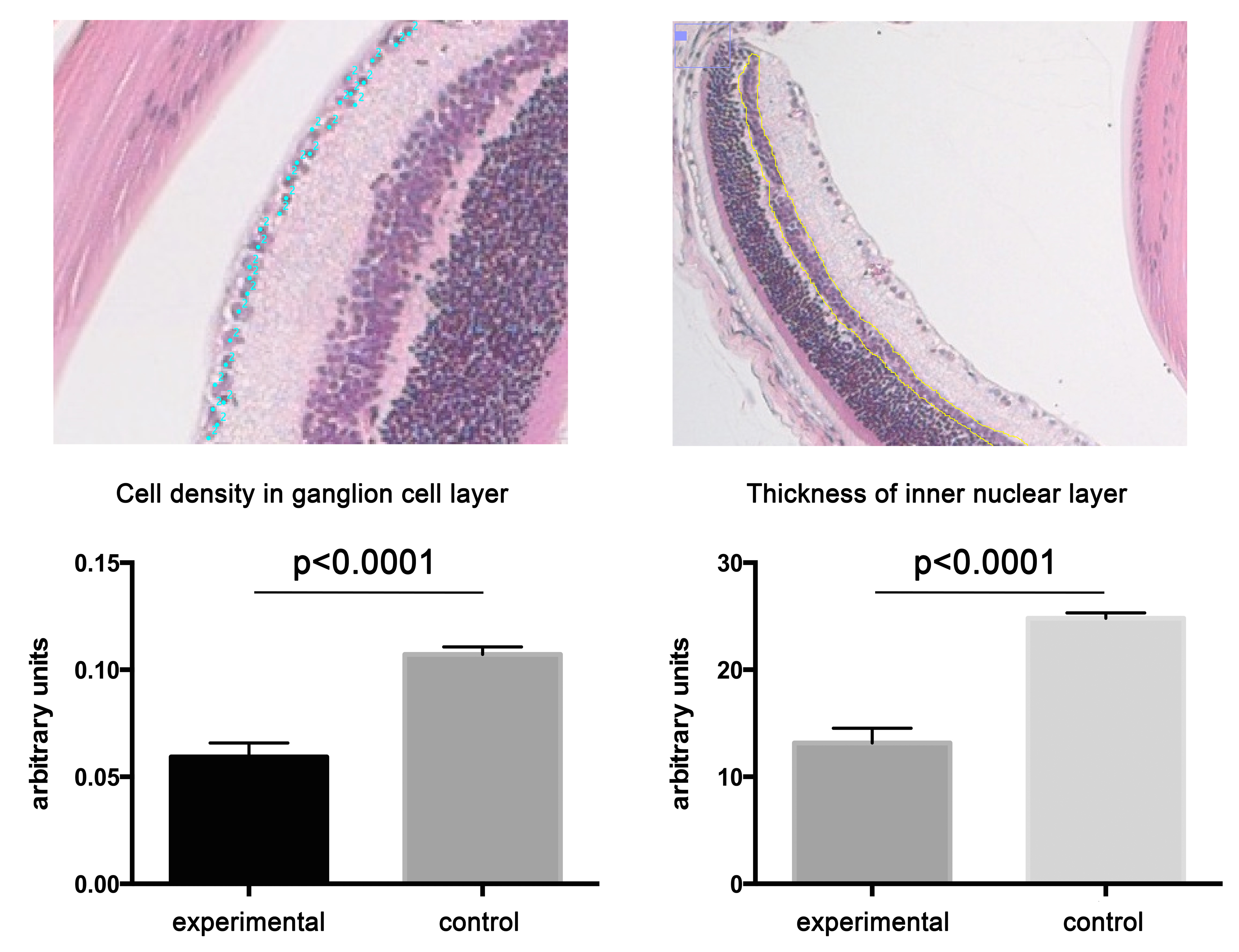

Supplement: S1 Fig — The reduction of the cell density in the ganglion cell layer was 45% (p < 0.0001; n = 6) compared to control eyes (n = 3). The decrease in thickness of the inner nuclear layer was of similar magnitude (47%, p < 0.0001). (TIF) [file pone.0119046.s001.tif]
